# Triple M Overlap Syndrome: Myocarditis, Myositis and Myasthenia Gravis After a Single Administration of Pembrolizumab

**DOI:** 10.7759/cureus.76834

**Published:** 2025-01-02

**Authors:** Carlota Baptista, Inês Margarido, Rita Bizarro, Francisco P Branco, Ana Faria

**Affiliations:** 1 Medical Oncology, Hospital Beatriz Ângelo, Loures, PRT; 2 Medical Oncology, Hospital da Luz, Lisbon, PRT

**Keywords:** immune checkpoint inhibitor-associated myasthenia, immune checkpoint inhibitor-associated myocarditis, immune checkpoint inhibitor-associated myositis, myositis-mg overlap, triple m syndrome

## Abstract

Triple M Overlap Syndrome (TMOS) is a rare and severe complication of immune checkpoint inhibitor (ICI) therapy, characterized by concurrent myocarditis, myositis and myasthenia gravis. There is a scarcity of evidence regarding this syndrome, underscoring the need for further research and reporting.

We report the case of a woman in her 70s with stage IV microsatellite instability-high (MSI-H) colon cancer who developed generalized myalgias and muscle weakness, along with bulbar and ocular symptoms, two weeks after the first administration of pembrolizumab. Laboratory tests revealed elevated creatine kinase, transaminase and troponin levels. Cardiac magnetic resonance imaging (MRI) findings were suggestive of myocarditis. Due to the constellation of myocarditis, myositis and myasthenia gravis, TMOS was diagnosed. Despite initial improvement with corticosteroid pulses, the patient’s clinical condition deteriorated, culminating in the need for mechanical ventilation due to respiratory failure. Sequential treatment with intravenous immunoglobulin (IVIG) and, ultimately, plasmapheresis resulted in gradual clinical improvement. After a prolonged Intensive Care Unit (ICU) stay, she was admitted to the medical ward, where she continued to improve with speech therapy and motor rehabilitation. ICI therapy was permanently discontinued.

A high level of suspicion is necessary for the diagnosis of this rare syndrome, and multidisciplinary collaboration is critical to optimize clinical outcomes. This case highlights the importance of evaluating myocarditis, myositis and myasthenia when any of them is diagnosed in a patient receiving ICIs. Prompt treatment initiation and ICI therapy discontinuation are crucial to maximizing the chances of a positive outcome.

## Introduction

Since its initial approval over 10 years ago, immune checkpoint inhibitor (ICI) therapy has transformed the treatment landscape of a wide range of malignancies, significantly improving treatment outcomes. ICIs block inhibitory receptors such as CTLA-4 and PD-(L)1, restoring T-cell activity and allowing the immune system to target and destroy cancer cells. Nonetheless, its use is associated with immune-related adverse events, which can affect nearly every organ and have a far more unpredictable timing than those related to chemotherapy. Management strategies are defined according to the grade of severity but typically involve ICI discontinuation and systemic corticosteroids as first-line interventions [1]. Triple M Overlap Syndrome (TMOS) is characterized by the co-existence of myocarditis, myositis and myasthenia gravis. It is an exceedingly rare complication of ICI therapy, with an in-hospital mortality rate approaching 60% [2]. Owing to its high mortality rate, suspicion of any one of these immune-related adverse events should lead to an assessment of all three [3-5].

We report a case of TMOS in an elderly patient with stage IV colon cancer after a single administration of pembrolizumab, emphasizing the importance of timely recognition and adequate intervention, in improving patient outcomes.

## Case presentation

We present the case of a woman in her 70s with stage IV microsatellite instability-high (MSI-H) colon cancer who was admitted to the Emergency Department two weeks after the first administration of pembrolizumab 200 mg, with complaints of generalized myalgias and muscle weakness over the past two days.

Past medical history includes mucinous adenocarcinoma of the ascending colon with lung metastases, which had recurred after primary treatment with surgery and adjuvant chemotherapy one year ago. She had no prior history of autoimmune or cardiac disease. Relevant surgical history includes cholecystectomy and hysterectomy. She did not take any chronic medications and had an Eastern Cooperative Oncology Group (ECOG) performance status of zero.

Physical examination at this time was unremarkable. The patient was afebrile, hemodynamically stable and had a peripheral oxygen saturation of 97% on room air. Exams at admission showed elevated transaminases, but were otherwise within normal range (Table 1); viral antigens were negative, and chest X-ray was unremarkable.

**Table 1:** Laboratory results at initial admission to the Emergency Department AST: aspartate aminotransferase; ALT: alanine aminotransferase; ALP: alkaline phosphatase; GGT: gamma-glutamyl transferase

Laboratory tests	Values	Normal range
Hemoglobin	13.6	12.0 - 15.2 g/dL
White cell count	4.85	3.90 - 11.10 x 10^9^/L
Neutrophils	3.35	1.80 - 7.40 x 10^9^/L
Platelets	146	170 - 430 x 10^9^/L
Urea	23	13 - 43 mg/dL
Creatinine	0.64	0.50 - 0.90 mg/dL
Total bilirubin	0.75	<2.00 mg/dL
AST	505	<40 UI/L
ALT	167	<41 UI/L
ALP	78	<105 UI/L
GGT	16	<38 UI/L
C-reactive protein	0.54	<0.50 mg/dL

The patient was discharged with analgesic medication as needed. She returned to the Emergency Department 48 hours later due to worsening myalgias, fatigue and muscle weakness, as well as new-onset diplopia. Notably, she had no cardiac symptoms. Physical examination revealed bilateral ptosis, external ophthalmoparesis with diplopia on lateroversion and proximal muscle weakness of the four limbs.

Blood tests at this time revealed elevated creatine kinase, aminotransferases, C-reactive protein and troponin (Table 2).

**Table 2:** Laboratory results on the second admission to the Emergency Department AST: aspartate aminotransferase; ALT: alanine aminotransferase; ALP: alkaline phosphatase; GGT: gamma-glutamyl transferase; CK: creatine kinase; hs-cTnI: high sensitivity cardiac troponin I

Laboratory tests	Values	Normal range
Hemoglobin	14.9	12.0 - 15.2 g/dL
White cell count	7.73	3.90 - 11.10 x 10^9^/L
Neutrophils	6.23	1.80 - 7.40 x 10^9^/L
Platelets	186	170 - 430 x 10^9^/L
Urea	33	13 - 43 mg/dL
Creatinine	0.85	0.50 - 0.90 mg/dL
Total bilirubin	0.57	<2.00 mg/dL
AST	1698	<40 UI/L
ALT	598	<41 UI/L
ALP	81	<105 UI/L
GGT	22	<38 UI/L
Amylase	40	22 - 80 UI/L
CK	37510	26 - 192 UI/L
hs-cTnI	2484	<58 ng/mL
C-reactive protein	6.40	<0.50 mg/dL

**Figure 1:**
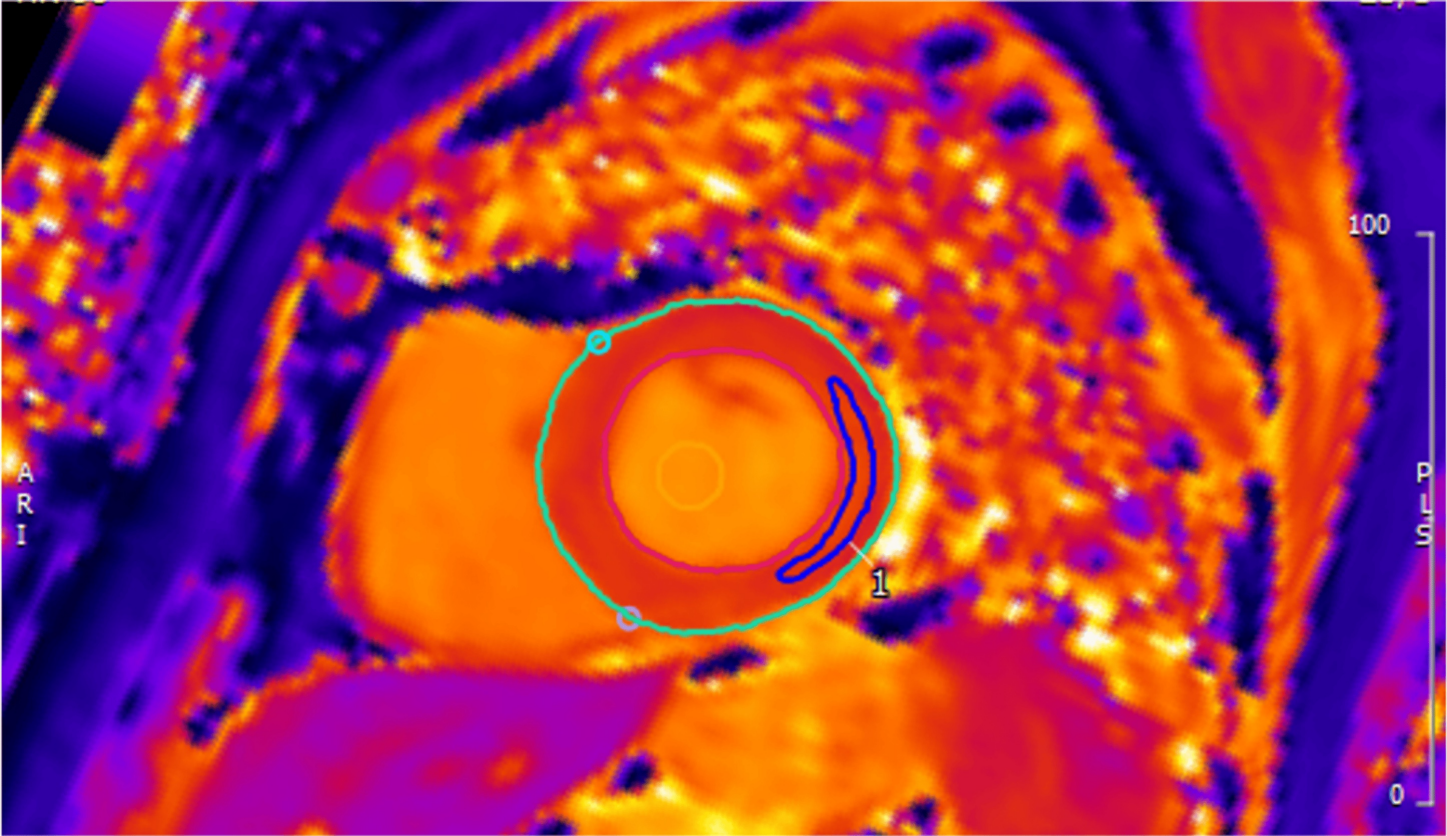
Cardiac MRI with native T1 mapping showing increased T1 MRI: magnetic resonance imaging

**Figure 2:**
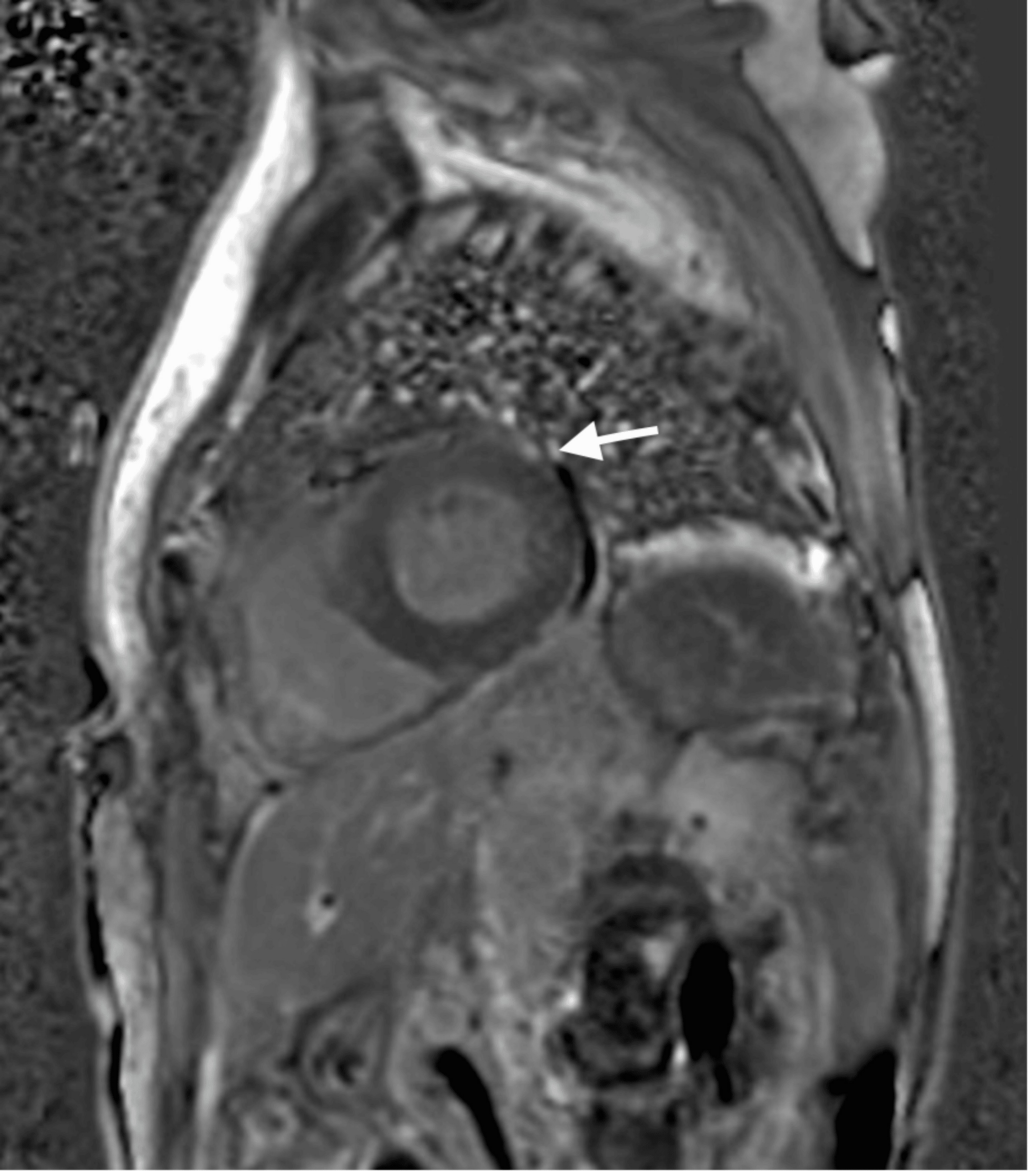
Late gadolinium enhancement cardiac MRI Cardiac MRI showing diffuse late gadolinium enhancement, predominantly in the basal and mid-cavity segments of the anterior and anteroseptal walls. These findings were less evident than usual as the patient had already been started on corticosteroids three days before. MRI: magnetic resonance imaging

The head computed tomography (CT) scan was unremarkable. Myositis and myocarditis secondary to ICI therapy was assumed, and the patient was started on intravenous methylprednisolone 500 mg daily. Electrocardiogram and telemetry monitoring showed no arrhythmias. Cardiac magnetic resonance imaging (MRI) showed increased native T1 mapping (Figure 1) and diffuse late gadolinium enhancement (Figure 2), suggestive of myocarditis.

Three days later, weakness and myalgia improved, troponin levels decreased and the patient was started on daily maintenance prednisolone (1 mg/kg). Due to persistence of ptosis and diplopia, a working diagnosis of myasthenia gravis was also considered, despite negative anti-acetylcholine receptor (AChR) antibodies and the patient was started on pyridostigmine.

Despite the initial improvement, three days later, the patient worsened, with new-onset dysphagia, dysarthria, dysphonia and dyspnea. She was transferred to the Intensive Care Unit (ICU), started on intravenous immunoglobulin (IVIG) and ventilated due to respiratory exhaustion. Plasmapheresis was started one week later due to the absence of improvement. The initial electromyography (EMG) at the time of ICU admission showed no abnormalities. However, a follow-up EMG two weeks later revealed findings consistent with diffuse muscle fiber injury, including fibrillation and slow denervation potentials at rest, as well as low-amplitude, short-duration, polyphasic motor units during contraction, suggesting the presence of active muscle necrosis.

She started to improve clinically, albeit slowly, requiring temporary tracheostomy and percutaneous endoscopic gastrostomy for ventilation and feeding, respectively. The patient was weaned off the ventilator 10 days after the last session of plasmapheresis. Continuous improvement was achieved through speech therapy, motor and pulmonary rehabilitation. The patient was transferred to a rehabilitation unit after being decannulated and able to feed by mouth, after a total of 93 days hospitalized. Pembrolizumab was permanently discontinued.

## Discussion

This case depicts the typical features of ICI-associated TMOS, in which myocarditis, myositis and myasthenia gravis present synchronously. The distinction between myositis and myasthenia gravis can be challenging due to overlapping manifestations seen in both conditions, namely ocular, bulbar and respiratory muscle weakness [4]. Our patient presented initially at the Emergency Department with generalized weakness and myalgias. Her symptoms quickly worsened over the following days without appropriate intervention, with ocular and bulbar involvement. Diagnostic workup for suspected immune-related myositis and myasthenia gravis should include blood testing for creatine kinase, aldolase, erythrocyte sedimentation rate, C-reactive protein and antibody testing (AChR, striational and muscle-specific tyrosine kinase) [3,4]. In this case, the patient presented with elevated creatine kinase, C-reactive protein, aspartate aminotransferase (AST) and alanine aminotransferase (ALT). Notably, the elevation of transaminases can result from non-hepatic sources, such as muscle injury, and may serve as a useful marker for myositis [3,6]. Indeed, transaminase elevation can present in patients undergoing ICI therapy and raise suspicion for hepatitis. Serum creatine kinase levels can be useful in distinguishing between hepatitis and myositis when the clinical scenario is unclear, as some patients with myositis can be otherwise asymptomatic [4]. Antibody testing is an important component of the myasthenia gravis diagnostic workup. Indeed, antibodies against the AChR can be negative in up to 15% of cases of myasthenia gravis, as observed in this patient [7]. Muscle-specific kinase (MuSK) antibodies could have provided additional information supporting this diagnosis.

The reported incidence of immune-related myocarditis is <1% but the mortality rate can be as high as 50% [1,8]. The timing of myocarditis presentation in this case was consistent with literature reports, typically developing within the first four cycles of treatment, although it can develop after a single administration [9,10]. Intravenous methylprednisolone 500-1000 mg daily for three days is suggested as initial therapy for immune-related myocarditis and admission to level two or three care facilities with electrocardiogram monitoring is recommended [1,3]. This approach was implemented in this case, leading to initial clinical improvement and a decrease in troponin levels. It is noteworthy that the patient’s subsequent clinical deterioration was largely attributable to the myositis and myasthenia, whereas the myocarditis responded favorably to corticosteroid pulses and there was no progression to overt heart failure. A paradoxical exacerbation of myasthenia gravis might occur when corticosteroids alone are used [11]. In a study by Safa et al., patients with immune-related myasthenia gravis who received upfront IVIG or plasmapheresis had a better outcome than patients who received corticosteroids alone [12]. Our patient was initially managed with corticosteroid pulses and IVIG and plasmapheresis were subsequently administered due to worsening bulbar and respiratory symptoms. Retrospectively, it is possible that upfront administration of IVIG and plasmapheresis might have prevented the progression of these symptoms.

Patients with myocarditis, myositis or myasthenia gravis, regardless of severity, should be promptly referred for specialist evaluation in order to maximize chances of a positive outcome [1,3,4].

Due to its rarity, reporting cases of TMOS is crucial to raise awareness within the medical community. Although oncologists and other specialists directly managing patients receiving ICIs are alert to the possibility of immune-related adverse events, these effects are less familiar to emergency physicians, who might initially come across these patients [13].

The positive outcome in this particular case, involving an elderly woman, underscores the importance of timely and appropriate treatment.

## Conclusions

TMOS, characterized by the concurrent development of myocarditis, myositis and myasthenia gravis, is a rare but severe complication of ICI therapy. This case emphasizes the importance of early recognition and prompt intervention to improve clinical outcomes. While myocarditis responded favorably to corticosteroid pulses, myositis and myasthenia required treatment escalation, including IVIG and plasmapheresis. This should lead to consideration of first-line IVIG or plasmapheresis in severe cases of immune-related myasthenia gravis. Due to its complexity and high fatality rate, it is crucial that healthcare providers maintain a high index of suspicion for TMOS in patients receiving ICI therapy.
